# Supplementary figures and images for: Prioritizing disease-related rare variants by integrating gene expression data
Source: PLoS Genet. 2024 Sep 30;20(9):e1011412. doi: 10.1371/journal.pgen.1011412 (PMC11466430; doi:10.1371/journal.pgen.1011412)

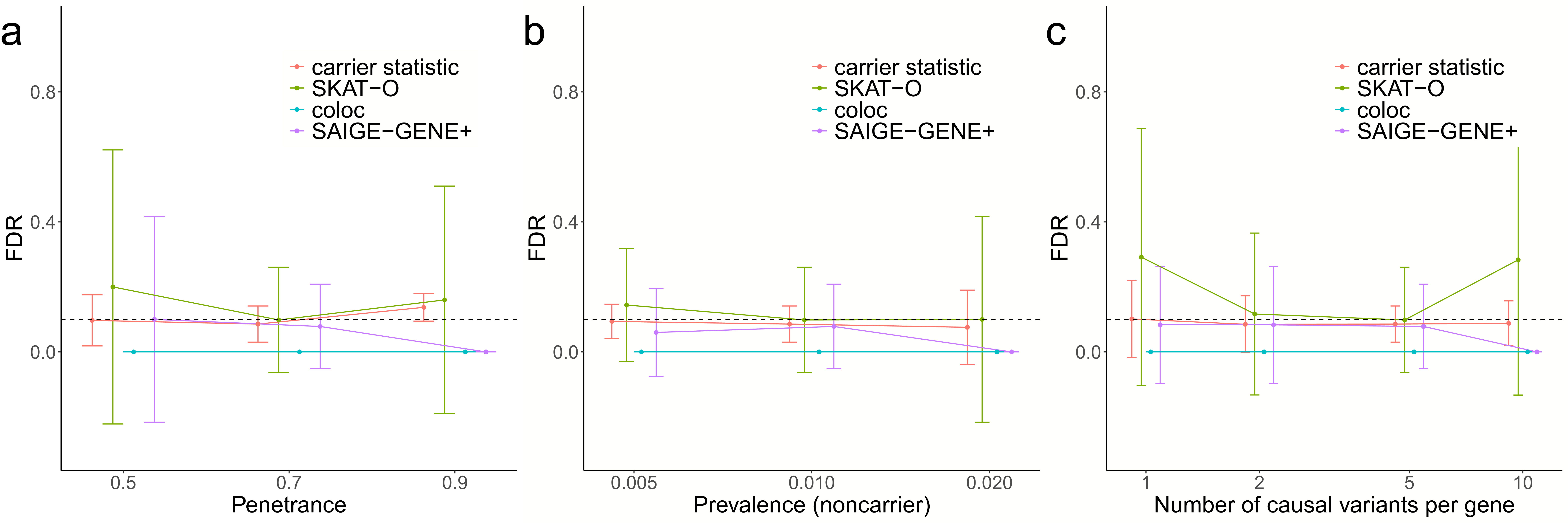

Supplement: S1 Fig — FDR for carrier statistic, SKAT-O, coloc, and SAIGE-GENE+ in simulations with varying (a) penetrance of causal variant, (b) prevalence in causal variant noncarriers, and (c) number of causal variants per causal gene. Error bar shows standard deviation across 100 simulation repeats. (TIF) [file pgen.1011412.s001.tif]

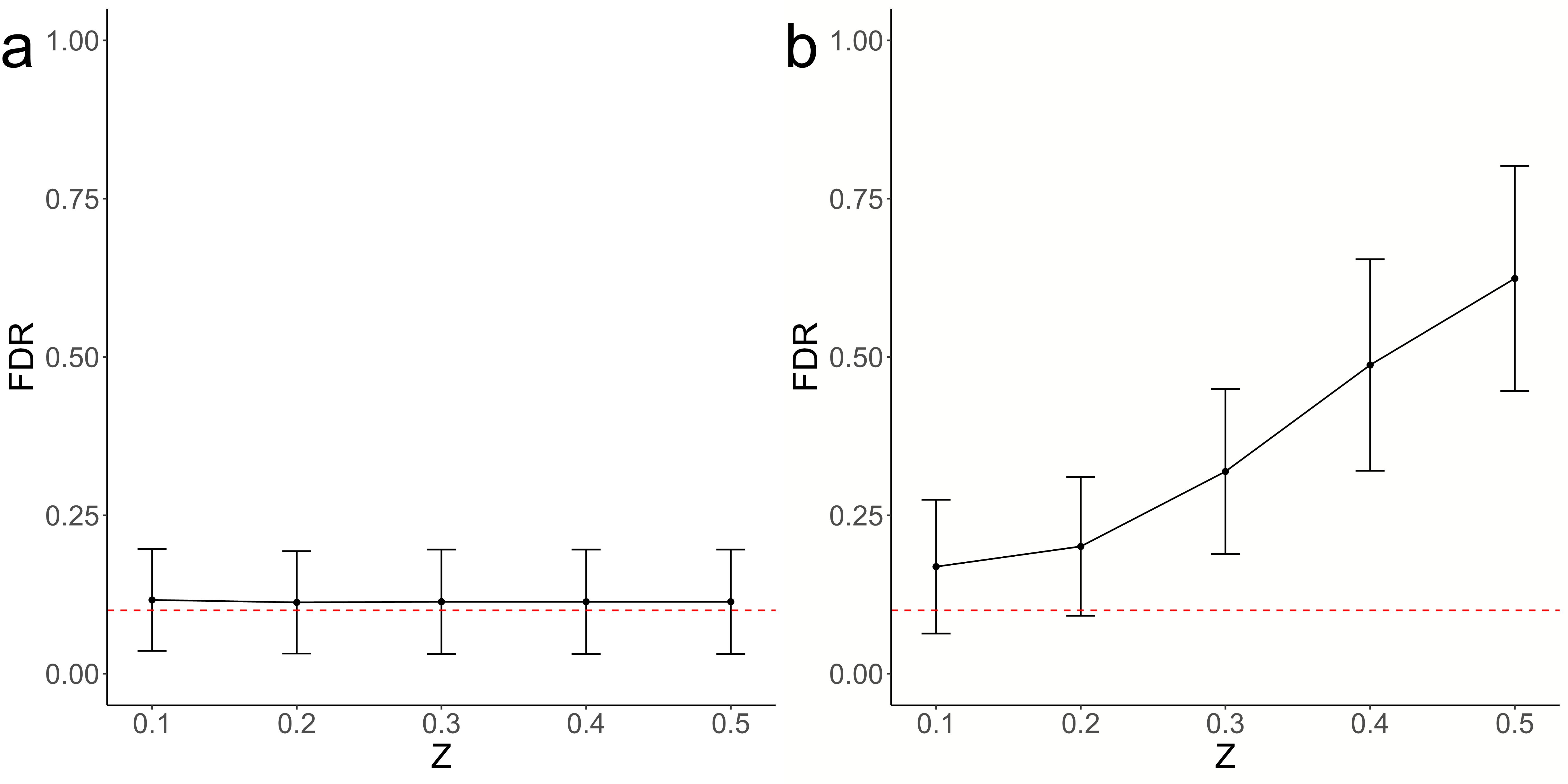

Supplement: S2 Fig — (a). FDR is well-calibrated for using gene expression from noncarriers in the same group of carriers as null distribution. (b). FDR showed substantial inflation for using gene expression from all noncarriers as null distribution. Z quantifies the level of systematic difference in the transcriptome between case group and control group. Error bar shows standard deviation across 100 simulation repeats. (TIF) [file pgen.1011412.s002.tif]

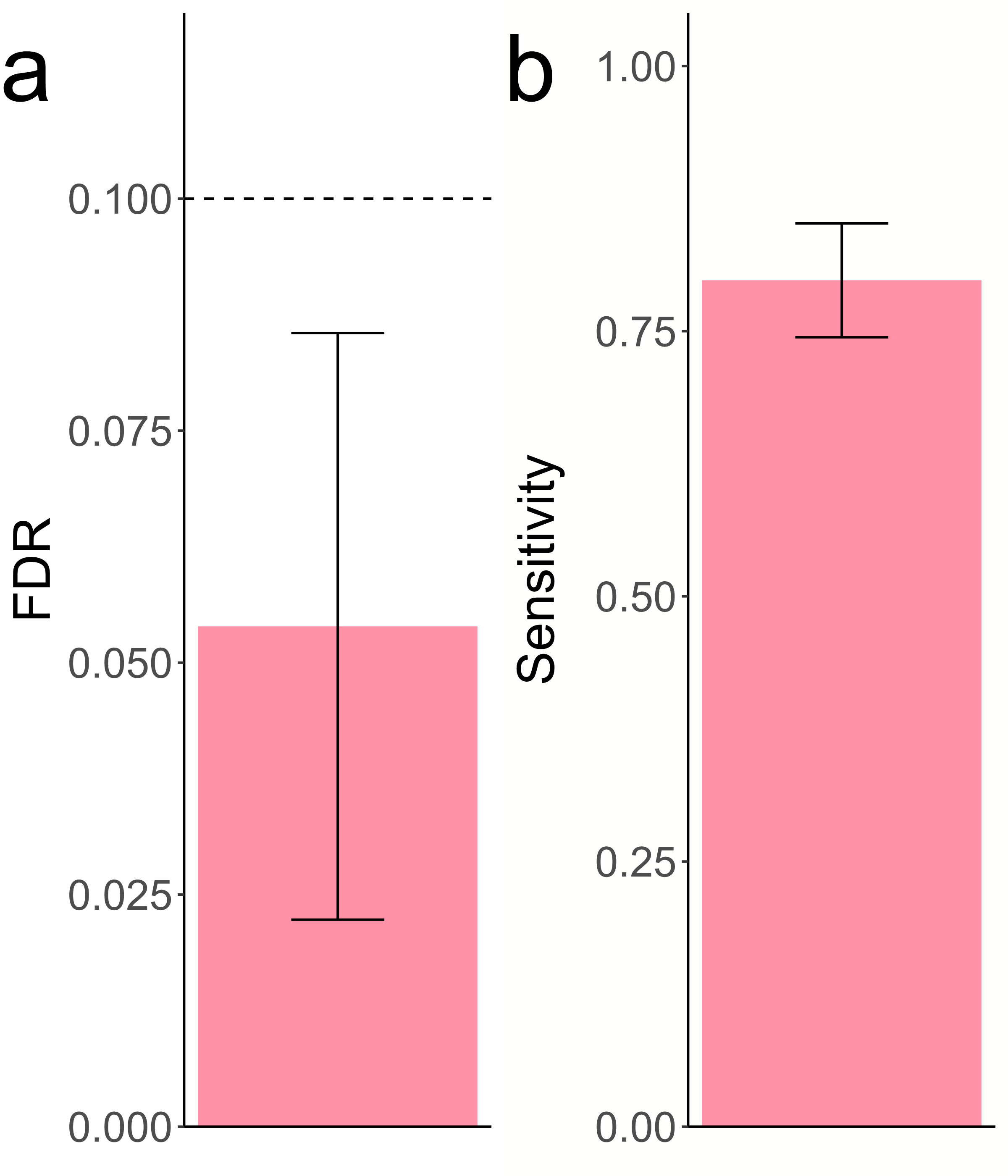

Supplement: S3 Fig — (a) FDR and (b) sensitivity of carrier statistic in simulations where both rare causal variants and rare protective variants are present. Error bar shows standard deviation across 100 simulation repeats. (TIF) [file pgen.1011412.s003.tif]

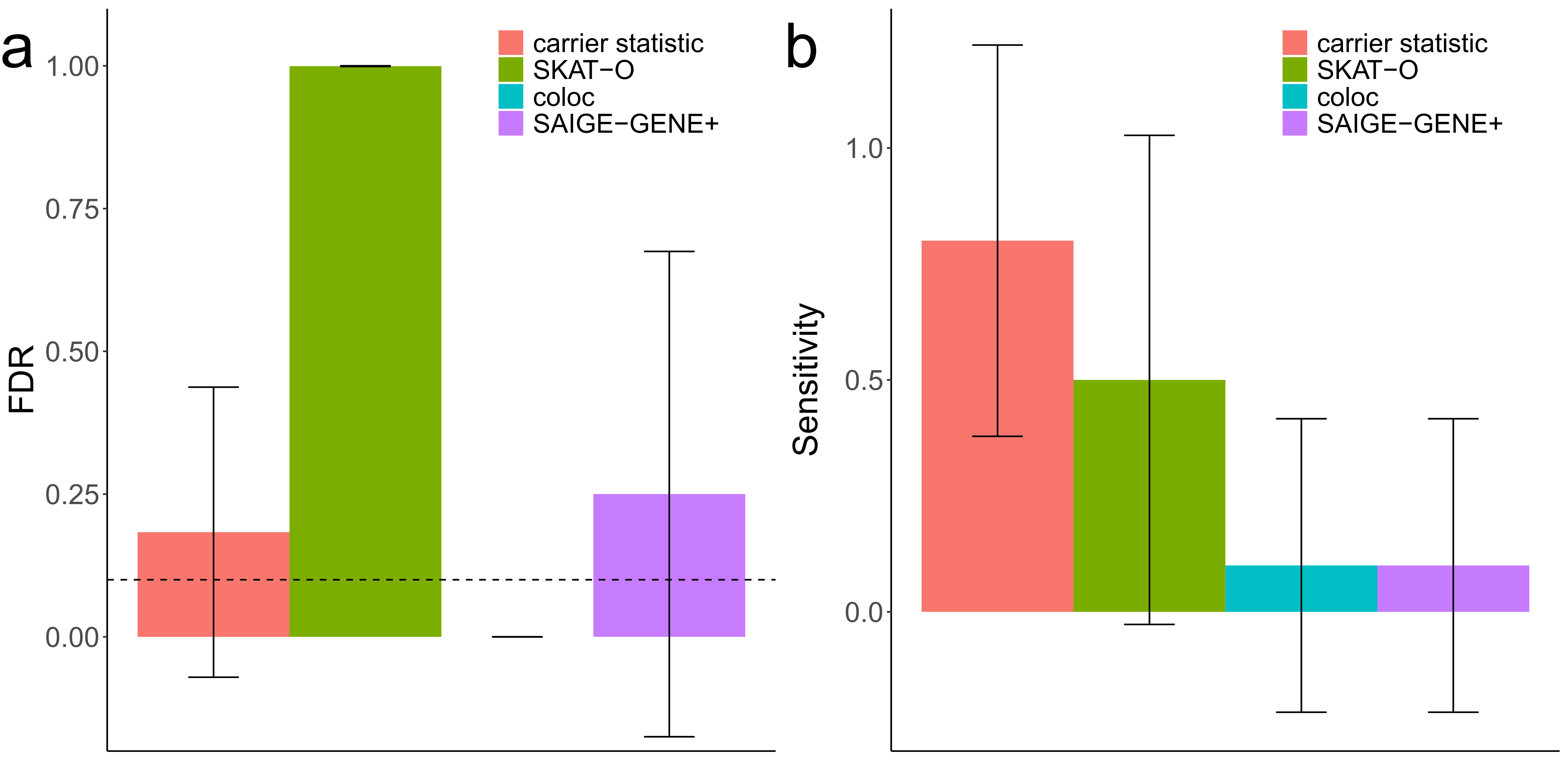

Supplement: S4 Fig — (a) FDR and (b) sensitivity of carrier statistic, SKAT-O, coloc, and SAIGE-GENE+ in simulations with only 1 causal gene and 1% case-control ratio. Error bar shows standard deviation across 100 simulation repeats. (TIF) [file pgen.1011412.s004.tif]

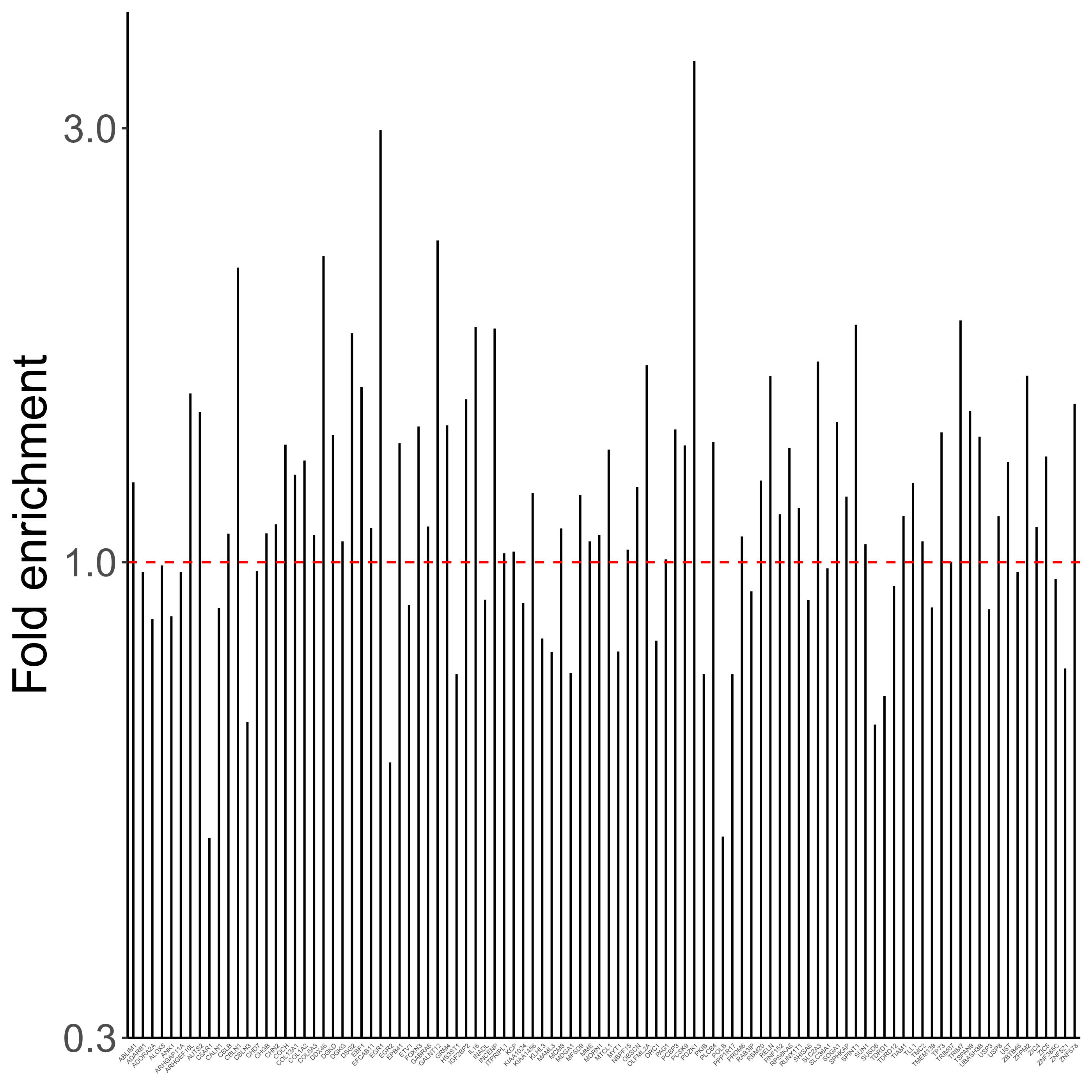

Supplement: S5 Fig — Genes were ranked according to decreasing order of carrier statistic. Fold enrichment was defined as the ratio of rare variants burden within the gene in case group compared to that in the control group. (TIF) [file pgen.1011412.s005.tif]
