# Supplementary material for: Prioritizing disease-related rare variants by integrating gene expression data
Source: PLoS Genet. 2024 Sep 30;20(9):e1011412. doi: 10.1371/journal.pgen.1011412 (PMC11466430; doi:10.1371/journal.pgen.1011412)
Supplement: Attachment — Submitted filename: Response.pdf [file pgen.1011412.s012.pdf]

PGENETICS-D-24-00550

Dear Dr Wong,
Thank you very much for submitting your Research Article entitled 'Prioritizing disease-related rare variants by integrating gene expression data' to PLOS Genetics. The manuscript was fully evaluated at the editorial level and by independent peer reviewers. The reviewers appreciated the attention to an important problem, but raised some concerns about the current manuscript. Based on the reviews, we will not be able to accept this version of the manuscript, but we would be willing to review a much-revised version. We cannot, of course, promise publication at that time. Should you decide to revise the manuscript for further consideration here, your revisions should address the specific points made by each reviewer. We will also require a detailed list of your responses to the review comments and a description of the changes you have made in the manuscript.

Response: We thank you for giving us the opportunity to improve our manuscript based on the referee comments and hope that the revised manuscript is now suitable for publication in PLOS Genetics.

Reviewers' Comments:

Reviewer #1: *Guo et al. proposed a carrier statistic to prioritize rare variants using gene expression data, with limited sample size. While it is a great attempt to solve a critical problem, there are a number of limitations.*

Response: Thank you for your insightful feedback. We appreciate your acknowledgment of the significance of our research problem and are pleased to address your valuable comments.

Major criticism 1. *The method needs more details. It is unclear how to compute the z score for each rare variant carrier. I assume carrier means an individual carrying the rare variant. I suggest write the definition using math formula explicitly. Similarly, the caption for Figure 1 requires elaboration, as the x-axis in Figure 1a is not clearly defined.*

Response: Thanks for the great suggestion. To formally define this concept, for each rare variant-gene pair, let $E_i^{carrier}$ $(i = 1, \dots, n^{carrier})$ and $E_j^{noncarrier}(j = 1, \dots, n^{noncarrier})$ represent the gene expression levels for carriers and noncarriers of that rare variant, respectively. Let $m^{noncarrier}$ and $\sigma^{noncarrier}$ denote the mean and standard deviation of gene expression among all rare variant noncarriers. Then the expression association z-score is computed as $\frac{1}{n^{carrier}}\sum_{i=1}^{n^{carrier}} \frac{E_i^{carrier}-m^{noncarrier}}{\sigma^{noncarrier}}$, referred to hereafter as carrier statistic. We have included the detailed mathematical definition in the **Methods: Carrier statistic** section.

Figure 1a in the main text illustrates a carrier statistic example for a rare variant-gene pair using violin plots. These plots depict gene expression levels among carriers and non-carriers of that variant, with each data point jittered along the x-axis to improve visualization. Therefore, the x-axis does not convey specific quantitative meaning. We have incorporated the detailed explanation into the caption of **Figure 1a** as suggested.

Major criticism 2. *The method requires both genotypes and gene expression for the same samples. This is very hard to achieve in practice, as the genotyped samples often differ from those available expression data. So the method application is very limited.*

Response: Thank you for raising this important point. It is indeed true that carrier statistic requires paired genotype and gene expression data. Fortunately, there already exist several large datasets that satisfy this condition, including the Genotype-Tissue Expression project (GTEx)[1], The Cancer Genome Atlas program (TCGA)[2], and PsychENCODE[3]. The Trans-Omics for Precision Medicine program (TOPMed)[4] is another significant resource, which has already produced over 100K WGS samples and is generating approximately 40K RNA-seq samples. TOPMed also offers a comprehensive range of multi-omics data, including 37K samples with metabolomics profiling, 57K samples with DNA methylation profiling, and 4K samples with proteomics assaying (more details are available at TOPMed's website, https://nhlbi.sph.umich.edu/omics/index.php). These

extensive datasets provide ample opportunities for applying our method and analyzing large-scale multi-omics data.

Our work underscores the promise of incorporating functional gene expression data into disease study, which allows quantitatively measuring the impact of rare variants that cannot be determined by looking at the variants alone. We hope that the results presented in this paper will serve to demonstrate the promise of the carrier statistic approach and will stimulate interest in the collection of both genotype and gene expression data for the same individuals in future case-control studies. This is especially crucial when the tissue relevant to the disease is accessible for analysis, such as in blood-related conditions, skin diseases, and certain types of cancer. Notably, when tissue samples are available, adding RNA-seq to a WGS-based GWAS will not substantially increase study costs. We anticipate that large sets of WGS and functional genomics data will be generated in the near future, and carrier statistics will be a useful method to dissect the molecular mechanism of some classes of diseases.

Major criticism 3. *The authors compared the methods with burden test, SKAT and SKAT-O, which do not incorporate expression information. So the comparison is unfair. It would be beneficial to compare the method with others that also utilize expression data. What about colocalization or similar methods?*

Response: As per your suggestion, we benchmarked the performance of carrier statistic using coloc[5], a method that estimates the posterior probability of colocalization between gene expression and GWAS association signals, to ensure a fair comparison. Genes with posterior probability of colocalization larger than 0.1 were determined as identified genes related to the disease. Here, a relatively loose threshold 0.1 was used instead of 0.5 suggested by the software to increase sensitivity of the coloc method. We also included SAIGE-GENE+[6], a method to perform variants collapsing association test for unbalanced case-control phenotypes, for comparison as suggested by **Reviewer 2** (Major criticism 4). We have excluded burden test and SKAT from our current comparisons, as SKAT-O, which implements a linear combination of burden test statistic and SKAT, demonstrated similar performance to these two methods in our initial simulation results.

While all four methods successfully controlled FDR at the nominal level (**Figure 1**), carrier statistic consistently achieved higher sensitivity compared to the other three methods (**Figure 2**), including coloc. We believe that the colocalization method is best suited for analyzing common SNPs with genome-wide significant association. In contrast, its statistical power substantially diminishes when applied to rare variants with less significant association p-values. This also highlights the advantage of carrier statistic, which is the first method that can effectively incorporate gene expression data to analyze rare variants associations.

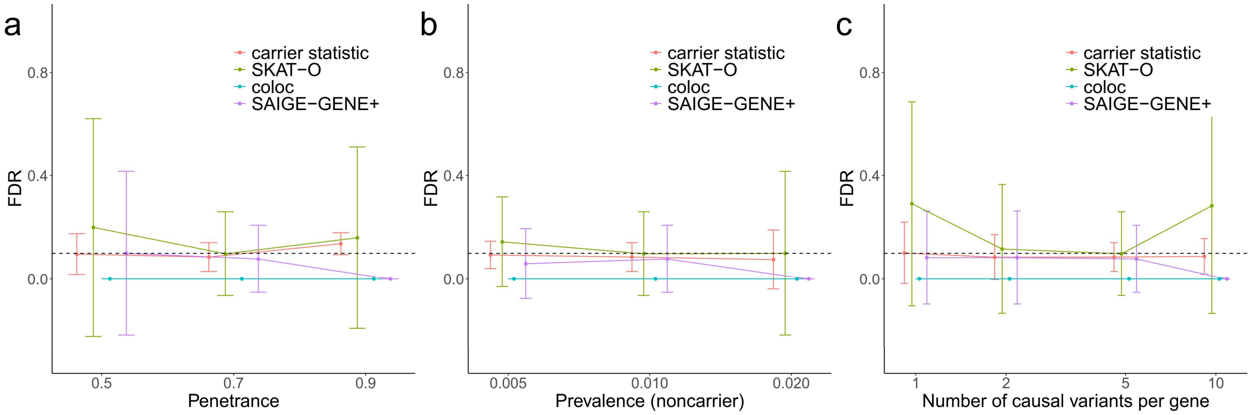

Figure 1. FDR for carrier statistic, SKAT-O, coloc, and SAIGE-GENE+ in simulations with varying (a) penetrance of causal variant, (b) prevalence in causal variant noncarriers, and (c) number of causal variants per causal gene. Error bar shows standard deviation across 100 simulation repeats.

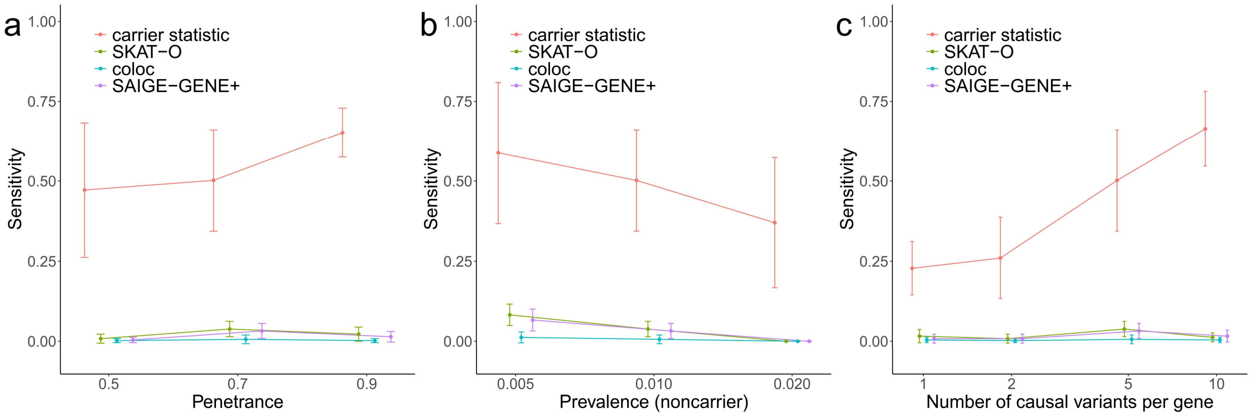

Figure 2. Carrier statistic achieves higher sensitivity than SKAT-O, coloc, and SAIGE-GENE+ in simulations with varying (a) penetrance of causal variant, (b) prevalence in causal variant noncarriers, and (c) number of causal variants per causal gene. Error bar shows standard deviation across 100 simulation repeats.

Major criticism 4. *In the Alzheimer application, the authors used FDR cutoff 0.2, which is quite high. Clarification on whether this threshold is recommended for the method, or if it was specifically chosen for this application, would be valuable.*

Response: Thanks for your question. In the real data application to Alzheimer's disease, we initially employed a traditional FDR cutoff of 0.05 and identified one significant gene, *COCH*, despite the constraints of a relatively small sample size consisting of 444 cases and 234 controls (totaling 678 individuals). Recognizing the inherent limitations in statistical power with this sample size, we subsequently increased the FDR cutoff to 0.2. This allowed us to enhance sensitivity and detect a broader set of potential gene associations, identifying a total of 15 genes that may be linked to Alzheimer's disease. In future applications to diseases with larger sample size, we recommend switching back to the traditional FDR cutoff of 0.05, which can minimize false positives and ensure robustness in scientific findings. For example, as detailed in the main text,

2,000 samples with 50% case-control ratio will be sufficient to achieve 80% sensitivity for large effect rare variants (Z = 4.5) at FDR cutoff 0.05. We have incorporated this into **Discussion** in the manuscript.

Reviewer #2: *In this manuscript, the authors present a statistical framework designed to prioritize disease-related rare variant-gene pairs. The authors provided simulations and real-data analysis to demonstrate the advantages of the carrier statistic. However, I believe several points could enhance the paper's quality and depth*.

In general, carrier statistic provides a novel framework for prioritizing disease-related rare variants and their regulated genes, achieving better statistical power especially when sample sizes are limited and multiple genes are considered. However, I believe that more methodological details about the carrier statistic and FDR control should be provided to make this statistical framework more solid.

Response: We are deeply grateful for your careful review of our manuscript and for your helpful and constructive feedback. We are happy to address your comments as follows.

Major criticism 1. *The authors defined the carrier statistic to quantify the degree to which the rare variant impacts gene expression level. However, the mathematical definition of this carrier statistic is unclear. I suggest providing more methodological details about the computation of the z-score mentioned in line 264.*

Response: Thanks for your great suggestion. To formally define this concept, for each rare variant-gene pair, let $E_i^{carrier}$ $(i = 1, \ldots, n^{carrier})$ and $E_j^{noncarrier}(j = 1, \ldots, n^{noncarrier})$ represent the gene expression levels for carriers and noncarriers of that rare variant, respectively. Let $m^{noncarrier}$ and $\sigma^{noncarrier}$ denote the mean and standard deviation of gene expression among all rare variant noncarriers. Then the expression association z-score is computed as $\frac{1}{n^{carrier}}\sum_{i=1}^{n^{carrier}}\frac{E_i^{carrier}-m^{noncarrier}}{\sigma^{noncarrier}}$, referred to hereafter as carrier statistic. We have included the detailed mathematical definition in the **Methods: Carrier statistic** section.

Major criticism 2. *In lines 89-91, the authors compared carrier statistics for disease-related variant-gene pairs with those for non-related pairs in the case group. However, Figure 1b appears to compare the density of carrier statistics between case and control groups. To verify the conclusion in the main text, it would be helpful to provide density plots of carrier statistic distributions for disease-related and non-related rare variant-gene pairs*.

Response: Thanks for pointing this out. We apologize for the lack of clarity in these sentences. Our intended message is that certain rare variants can significantly alter the expression of disease-related genes and move the expression levels towards the extreme and abnormal ranges, leading to elevated disease risk. While rare variants that cause extreme expression levels for genes unrelated to the disease may also exist in the control group, they are uncommon. Consequently, the distribution of carrier statistics in the case group will exhibit a heavier tail compared to that in the control group. We have revised and updated the expression in the main text accordingly.

Major criticism 3. *The authors proposed a method to control the false discovery rate (FDR) in lines 274-279. I believe it is necessary to explain why the FDR can be computed in this way. Is it possible that disease-related rare variant-gene pairs also exist in the control group? For instance, there might be protective rare variant-gene pairs with large carrier statistics that only occur in the control group*.

Response: This is an excellent point. It has been suggested that rare variants can be either deleterious or protective, and that variance component-based methods are more powerful than burden-type method for rare variants association studies, as they account for the different directions of variant effects. While variants collapsing methods evaluate the enrichment of deleterious variants burden in cases and enrichment of protective variants burden in controls, our method specifically assesses the enrichment of tail distribution of carrier statistics in cases. The rationale behind this approach is that deleterious variants are expected to significantly affect gene expression, whereas protective variants are more likely to maintain gene expression levels within a normal range. Consequently, extreme values for carrier statistics will be enriched in the case group, reflecting those rare deleterious variants that cause disease.

To further address this, we conducted additional simulations to evaluate the performance of carrier statistic in the presence of both deleterious, rare causal variants and rare protective variants. In these simulations, we randomly selected 50 causal genes and 50 protective genes, each containing 5 rare causal variants or 5 protective variants. Gene expression among rare variant carriers was perturbed by $z * sd$ fold, where z was set to 5 for rare causal variants and 2.5 for rare protective variants, with sd representing the standard deviation of normalized gene expression in the reference dataset. Following this, we simulated the disease status, assuming a penetrance of $p_{carrier} = 0.7$ for causal variants, a genotypic relative risk of $GRR_{protective} = 0.1$ for protective variants (presence of a protective variant will decrease disease risk by tenfold), and a prevalence of $p_{noncarrier} = 0.01$ for non-carriers of both types. Due to low disease prevalence, protective variants are unlikely to be observed in the control group, and their presence in the case group is even less probable as protective variants imply a lower risk of disease. Therefore, we adjusted the population MAF for protective variants to ensure their representation in the control group. Specifically, setting the population MAF for protective variants to 0.1% led to an observed frequency in the control group similar to that of causal variants in the case group (**Table 1**). Under these simulation conditions, carrier statistic still effectively controlled FDR at the nominal level (**Figure 3a**) and achieved over 75% sensitivity in identifying causal genes (**Figure 3b**). These results indicate that the presence of protective variants will not affect the validity or performance of our method.

Table 1. Rare variants burden in simulated case-control data when population MAF of protective variants is 0.1%.

	Causal variant	Protective variants
Case	200.3	34.3
Control	2.9	243.4

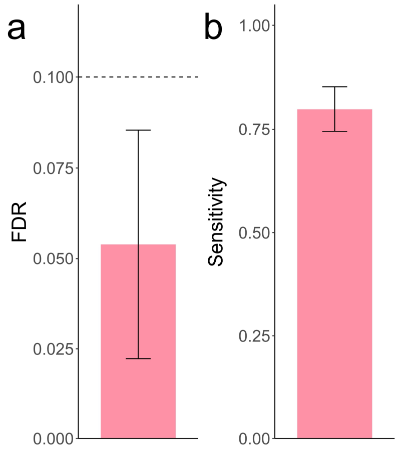

Figure 3. (a) FDR and (b) sensitivity of carrier statistic in simulations where both rare causal variants and rare protective variants are present. Error bar shows standard deviation across 100 simulation repeats.

Major criticism 4. *In the simulation, the authors compared carrier statistic with burden test, SKAT and SKAT-O. However, burden test and SKAT are designed to examine the association between one region and the trait of interest. It would be beneficial to compare carrier statistic with other methods in an additional simulation setting with only one causal gene (instead of m=50 in the current setting) and a smaller prevalence (like 1% in the SKAT simulation setting). Besides, carrier statistic should also be compared with SAIGE-GENE+ (Zhou, Wei, et al. 2022), which is developed to conduct variance component set-based tests for unbalanced case-control phenotypes*.

Response: Thanks for your valuable suggestion. In response, we benchmarked the performance of carrier statistic using SAIGE-GENE+[6], a method designed for variants collapsing association test with unbalanced case-control phenotypes. We also included coloc[5], a method that estimates the posterior probability of colocalization between gene expression and GWAS association signals, as suggested by **Reviewer 1** (Major criticism 3). Genes with a posterior probability of colocalization larger than 0.1 were identified as potentially related to the disease. Here, we used this lower threshold of 0.1 instead of the default 0.5 to increase sensitivity of the coloc method. We have excluded burden test and SKAT from our current comparisons, as SKAT-O, which implements a linear combination of burden test statistic and SKAT, demonstrated similar performance to these two methods in our initial simulation results.

Our updated simulations revealed that all four methods controlled FDR at the nominal level (**Figure 1**), with the carrier statistic demonstrating consistently higher sensitivity compared to the other three methods (**Figure 2**), including SAIGE-GENE+.

As per your request, we also performed additional simulation setting with only one causal gene. Given that we had set the prevalence in the general population to 1% in previous simulations, we interpreted your comment as a request to assess the performance of the carrier

statistic in scenarios with highly unbalanced case-control ratios (i.e., low prevalence in the case-control data). We simulated 500 cases and 50,000 controls, reflecting a 1% case-control ratio. Under these conditions, carrier statistic maintained well-calibrated FDR (**Figure 4a**) and showed substantially higher sensitivity than other three methods (**Figure 4b**). Of note, SKAT-O produced a significant proportion of false positives with unbalanced case-control ratio, a finding also reported in the SAIGE-GENE+ paper[6]. SAIGE-GENE+ controlled FDR effectively but showed low sensitivity, possibly due to the small number of case samples that can be attributed to the causal variants in any gene region, as discussed in the main text.

Taken together, these results indicate that carrier statistic performs robustly when only one causal gene is present and case-control ratio is highly unbalanced.

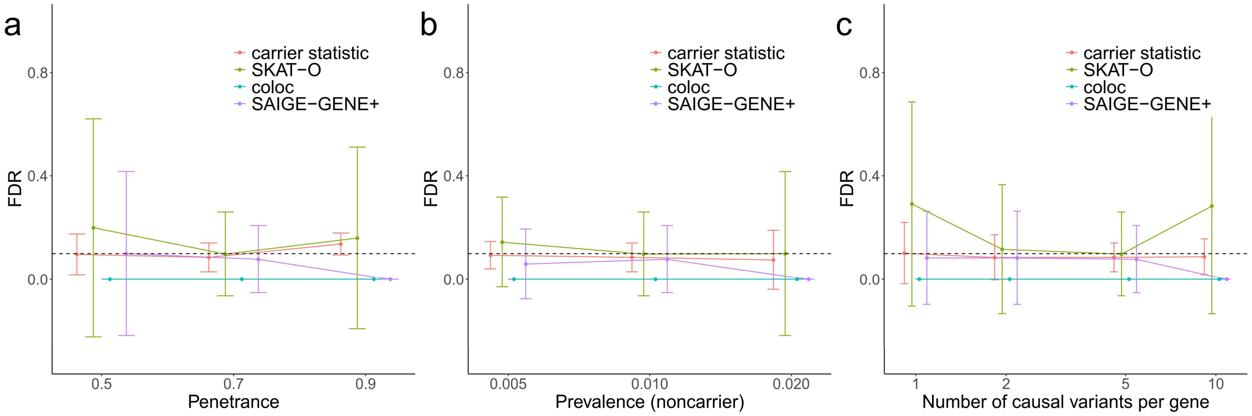

Figure 1. FDR for carrier statistic, SKAT-O, coloc, and SAIGE-GENE+ in simulations with varying (a) penetrance of causal variant, (b) prevalence in causal variant noncarriers, and (c) number of causal variants per causal gene. Error bar shows standard deviation across 100 simulation repeats.

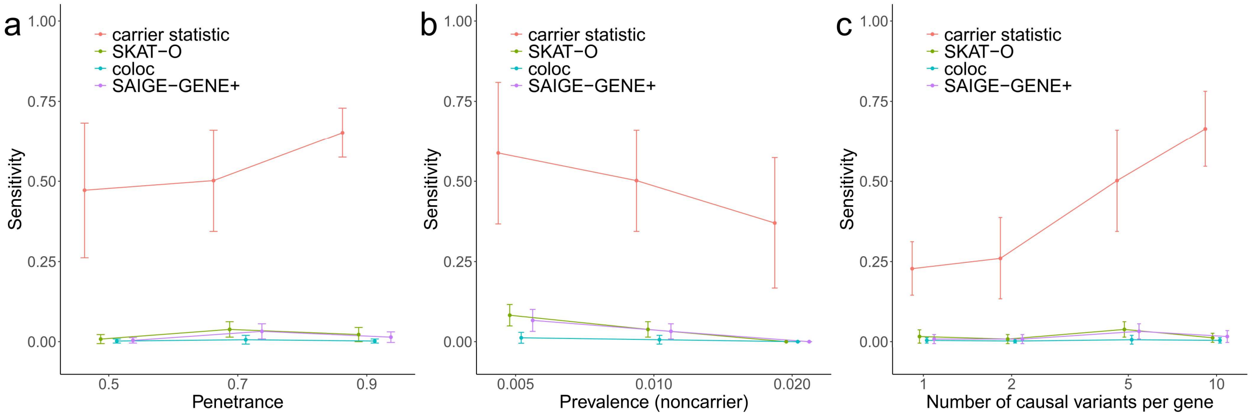

Figure 2. Carrier statistic achieves higher sensitivity than SKAT-O, coloc, and SAIGE-GENE+ in simulations with varying (a) penetrance of causal variant, (b) prevalence in causal variant noncarriers, and (c) number of causal variants per causal gene. Error bar shows standard deviation across 100 simulation repeats.

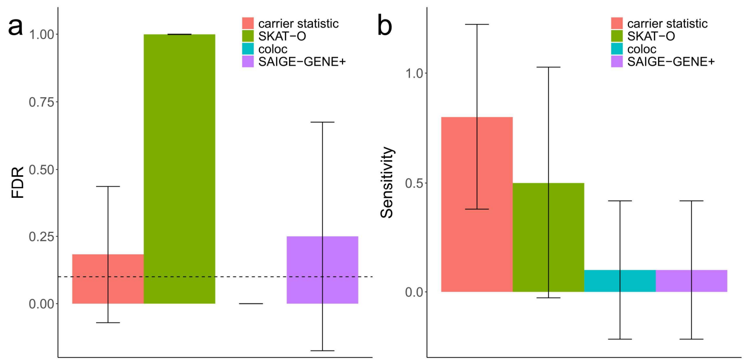

Figure 4. (a) FDR and (b) sensitivity of carrier statistic, SKAT-O, coloc, and SAIGE-GENE+ in simulations with only 1 causal gene and 1% case-control ratio. Error bar shows standard deviation across 100 simulation repeats.

Major criticism 5. *The range of MAF for causal rare variants simulated in line 291 should be provided*.

Response: The minor allele count (MAC) for rare causal variants spans from 1 to 125, with the corresponding MAF ranging from 0.0004% to 0.05%. We have integrated this information into the revised manuscript as per your suggestion.

Major criticism 6. *Related to comment (3), in real data applications with unbalanced case-control ratios (like the Alzheimer's disease study), how to achieve a good FDR control to distinguish disease-related rare variant-gene pairs from non-related pairs with large carrier statistic by chance in the case group*?

Response: Thanks for your question. In the main text, we applied our method to Alzheimer's disease data from four aging cohort studies—ROS, MAP, MSBB, and the Mayo Clinic—comprising 444 cases and 234 controls. This results in a relatively balanced case-control ratio of approximately 1.9. In general, one can combine multiple cohorts with both WGS and gene expression data to increase total sample size, often from healthy individuals, leading to more control samples and a potential imbalance in the case-control ratio. To address this, we have performed additional simulations demonstrating that the carrier statistic can still effectively identify underlying causal genes while maintaining well-calibrated FDR, as shown in **Figure 4**. Consequently, we anticipate that the carrier statistic will be a valuable tool for producing novel and scientifically valid results in large-scale population studies for complex diseases, particularly as multi-omics and genome sequencing data become more prevalent now.

References

1. Consortium, G. The GTEx Consortium atlas of genetic regulatory effects across human tissues. *Science* **369**, 1318-1330 (2020).
2. Tomczak, K., Czerwińska, P. & Wiznerowicz, M. Review The Cancer Genome Atlas (TCGA): an immeasurable source of knowledge. *Contemporary Oncology/Współczesna Onkologia* **2015**, 68-77 (2015).
3. Gandal, M.J. *et al.* Transcriptome-wide isoform-level dysregulation in ASD, schizophrenia, and bipolar disorder. *Science* **362**(2018).
4. Taliun, D. *et al.* Sequencing of 53,831 diverse genomes from the NHLBI TOPMed Program. *Nature* **590**, 290-299 (2021).
5. Wallace, C. A more accurate method for colocalisation analysis allowing for multiple causal variants. *PLoS genetics* **17**, e1009440 (2021).
6. Zhou, W. *et al.* SAIGE-GENE+ improves the efficiency and accuracy of set-based rare variant association tests. *Nature genetics* **54**, 1466-1469 (2022).
