# Supplementary material for: Prioritizing disease-related rare variants by integrating gene expression data
Source: PLoS Genet. 2024 Sep 30;20(9):e1011412. doi: 10.1371/journal.pgen.1011412 (PMC11466430; doi:10.1371/journal.pgen.1011412)
Supplement: Attachment — Submitted filename: Review.docx [file pgen.1011412.s011.docx]

In this manuscript, the authors present a statistical framework designed to prioritize disease-related rare variant-gene pairs. The authors provided simulations and real-data analysis to demonstrate the advantages of the carrier statistic. However, I believe several points could enhance the paper’s quality and depth:

(1) The authors defined the carrier statistic to quantify the degree to which the rare variant impacts gene expression level. However, the mathematical definition of this carrier statistic is unclear. I suggest providing more methodological details about the computation of the z-score mentioned in line 264.
(2) In lines 89-91, the authors compared carrier statistics for disease-related variant-gene pairs with those for non-related pairs in the case group. However, Figure 1b appears to compare the density of carrier statistics between case and control groups. To verify the conclusion in the main text, it would be helpful to provide density plots of carrier statistic distributions for disease-related and non-related rare variant-gene pairs.
(3) The authors proposed a method to control the false discovery rate (FDR) in lines 274-279. I believe it is necessary to explain why the FDR can be computed in this way. Is it possible that disease-related rare variant-gene pairs also exist in the control group? For instance, there might be protective rare variant-gene pairs with large carrier statistics that only occur in the control group.
(4) In the simulation, the authors compared carrier statistic with burden test, SKAT and SKAT-O. However, burden test and SKAT are designed to examine the association between one region and the trait of interest. It would be beneficial to compare carrier statistic with other methods in an additional simulation setting with only one causal gene (instead of m=50 in the current setting) and a smaller prevalence (like 1% in the SKAT simulation setting). Besides, carrier statistic should also be compared with SAIGE-GENE+ (Zhou, Wei, et al. 2022), which is developed to conduct variance component set-based tests for unbalanced case-control phenotypes.
(5) The range of MAF for causal rare variants simulated in line 291 should be provided.
(6) Related to comment (3), in real data applications with unbalanced case-control ratios (like the Alzheimer’s disease study), how to achieve a good FDR control to distinguish disease-related rare variant-gene pairs from non-related pairs with large carrier statistic by chance in the case group?

In general, carrier statistic provides a novel framework for prioritizing disease-related rare variants and their regulated genes, achieving better statistical power especially when sample sizes are limited and multiple genes are considered. However, I believe that more methodological details about the carrier statistic and FDR control should be provided to make this statistical framework more solid.
